# The Impacts of Therapeutic Interventions on Reducing Aggression Among Forensic Psychiatric Inpatients: A Scoping Review

**DOI:** 10.1192/bjo.2026.11090

**Published:** 2026-06-30

**Authors:** Margret Lo, Leen Elshikh, Mark Kaggwa, Britta Ostermeyer, Andrew Olagunju

**Affiliations:** 1 McMaster University, Hamilton, Canada; 2 Oklahoma University, Oklahoma, USA

## Abstract

**Aims:**

Aggression is prevalent in forensic psychiatric inpatient settings and poses significant safety risks to both patients and staff. A variety of interventions have been implemented to manage aggressive behaviour; however, no study has comprehensively synthesized and clearly summarized the existing evidence on these interventions. This scoping review aimed to identify and map the therapeutic interventions used to mitigate aggression among forensic psychiatric inpatients and to summarize the reported outcomes of these interventions.

**Methods:**

This review was conducted in accordance with the Joanna Briggs Institute (JBI) methodology for scoping reviews and reported following the PRISMA-ScR guidelines. Database search was performed in MEDLINE, Embase, PsycINFO, Cochrane CENTRAL, EMCARE, and Evidence-Based Medicine (EBM) Reviews, without restrictions on date or language to identify eligible reports. Study selection, data extraction, and quality assessment were performed independently by at least two reviewers.

**Results:**

Of the 1375 records identified, 58 studies were included in the final review. Psychological and behavioural interventions comprised nearly two-thirds of included studies and were generally associated with consistently positive outcomes. Approximately, 75% of studies reported reductions in the frequency and/or severity of aggression, most commonly assessed using observational measures. Interventions that were both staff- and patient-centred demonstrated the most favourable effects. However, substantial methodological heterogeneity and incomplete reporting limited comparability across studies.

**Conclusion:**

Evidence suggests that structured, multi-component therapeutic interventions are beneficial in reducing aggression among forensic psychiatric inpatients. Future research should emphasize standardized outcome measures and more rigorous comparative study designs are needed to improve the clinical applicability of findings.

